# Racial disparities in young-onset patients with colorectal, breast and testicular cancer

**DOI:** 10.7150/jca.32435

**Published:** 2019-08-29

**Authors:** Jingjing Wu, Jianzhong Ye, Wenrui Wu, Daiqiong Fang, Kaicen Wang, Liya Yang, Xianwan Jiang, Qiangqiang Wang, Lanjuan Li

**Affiliations:** 1 State Key Laboratory for Diagnosis and Treatment of Infectious Diseases, The First Affiliated Hospital, School of Medicine, Zhejiang University, Hangzhou, China; 2 Collaborative Innovation Center for Diagnosis and Treatment of Infectious Diseases, Hangzhou, China

**Keywords:** survival analysis, racial disparities, colorectal cancer, breast cancer, testicular cancer

## Abstract

**Aims**: Racial disparities in cancer mortality persist despite rapid developments in cancer treatment strategies. In recent decades, an increased frequency of patients with young-onset cancer has been reported. However, few studies have assessed racial disparities in clinical features and overall survival among young-onset patients with colorectal, breast, and testicular cancer. Therefore, we evaluated racial disparities in cancer mortality for these three cancer types.

**Methods**: We extracted the data of eligible patients from the Surveillance, Epidemiology and End Results (SEER) database from 1973 to 2014. Overall and cancer-specific survival rates were compared among races using Kaplan-Meier curves. Adjusted hazard ratios (HRs) with 95% confidence intervals (CIs) were calculated, and the association of race with survival was influenced by marital status, surgery and disease stage in Cox proportional hazard models.

**Results**: We collected the data of 19,574 patients with colorectal cancer, 68,733 with breast cancer, and 26,410 with testicular cancer; all were aged 25-40 years. A higher proportion of Blacks presented with a distant stage at diagnosis compared to Whites and Others (colorectal cancer: 18.0%, 18.5% and 18.4%, respectively, P = 0.004; breast cancer: 3.5%, 6.3% and 4.0%, respectively, P < 0.001; testicular cancer: 6.9%, 10.8% and 8.6%, respectively, P < 0.001). Multivariate analysis showed that Blacks had the highest overall mortality rate (colorectal cancer, HR, 1.277, 95% CI: 1.198, 1.361, P < 0.001; breast cancer, HR, 1.471, 95% CI: 1.420, 1.525, P < 0.001; testicular cancer, HR, 1.887, 95% CI: 1.562, 2.281, P < 0.001). In stratified analyses, Unmarried Blacks had a higher mortality rates (colorectal cancer, HR, 1.318, 95% CI: 1.211, 1.435, P < 0.001; breast cancer, HR, 1.465, 95% CI: 1.394, 1.541, P < 0.001; testicular cancer, HR, 1.944, 95% CI: 1.544, 2.447, P < 0.001). Furthermore, Blacks with colorectal and breast cancer had a higher risk of mortality than Whites at every disease stage, with greatest disparities occurred among individuals at localized stage. The influence of racial disparities on survival was consistent among patients who accepted surgery, but was weak among those who did not undergo surgery for colorectal cancer (Blacks, HR, 1.027, 95% CI: 0.866, 1.219, P = 0.758; Others, HR, 0.919, 95% CI: 0.760, 1.112, P = 0.386) and testicular cancer (Blacks, HR, 1.039, 95% CI: 0.538, 2.007, P = 0.909; Others, HR, 0.772, 95% CI: 0.388, 1.533, P = 0.459).

**Conclusions**: We demonstrated that Blacks had a worse prognosis for young-onset colorectal, breast, and testicular cancer. Marital status, cancer-directed surgery and disease stage may influence the association of race with the risk of mortality. Equal access to high-quality medical care among races, greater social support and comprehensive interventions are required. Moreover, further studies need to clarify the effects of biological properties like genetic differences between races on cancer patient survival.

## Introduction

Solid tumours remain the leading cause of death worldwide 1. Colorectal, breast and testicular cancer are commonly diagnosed in both young women and men 2-5. Significant improvements have been made in survival outcomes, attributed to rapid advances in the implementation of routine cancer screening, molecular characterisation and treatment strategies for the major cancer types 6, 7. There is a worrying trend toward an increase in the rate of young-onset cancer 8-11. Young patients are more likely than their older counterparts to present with regional or distant disease. Some studies illustrated that young-onset patients were recognized to be more aggressive compared with counterparts 12, 13. Yet, it remains to be controversial. Others have demonstrated improved disease-specific survival in young-onset patients compared with individuals diagnosed at older ages.

A disparity in survival rates between Black and White patients has been reported for various kinds of cancer. Black and African-American patients have poor overall survival (OS) for a variety of tumours 14. The underlying mechanism of this persistent racial disparity remains unclear. Differences in the aggressiveness of diseases characterised by undifferentiated and distant metastasis are likely involved 15. Lower rates of surgical treatment in Blacks have only been partially explained, as surgical treatment considerably improves the survival rates for most cancers 16.

Study of patients with young-onset cancer offers an opportunity to examine differences in survival by race and minimizes the potential impact that cancer screening among individuals younger than 40 years old. Few studies have rigorously compared survival differences among young patients of different races with colorectal, breast and testicular cancer 16. Considering the longer life expectancy of younger patients, it is critical to understand and characterise the mechanism underlying the racial disparities in survival outcomes, to inform appropriate surveillance strategies for these populations. Meanwhile, most race/ethnicity-oriented studies focused on investigation of socioeconomic status to explain the racial disparities in cancer patient survival. Less is known whether the influence of racial disparities consistent among marital status, disease stage and surgery. In this population-based study, we furtherly investigated the influence of marital status, surgery and disease stage on the association between race and the prognoses of colorectal, breast and testicular cancer.

## Methods

### Data source

We used data from the Surveillance, Epidemiology and End Results (SEER) 18 registry database. The SEER 18 registries, which cover approximately 27.8% of the US population, include all incident cancer cases diagnosed in the following SEER cancer registries: Alaskan Natives, Atlanta, Georgia; Connecticut; Detroit, Michigan; Hawaii; Iowa; New Mexico; San-Francisco Oakland, California; Seattle, Washington; Utah; Los Angeles, California; San Jose-Monterey, California; rural Georgia; Greater California, Kentucky, Louisiana, Greater Georgia, and New Jersey. The SEER database contains high-quality demographic information tabulated by age, sex, race/ethnicity, year of diagnosis, and geographic region. All data obtained from the SEER database are freely available; approval from an institutional review board or ethics committee was not required for use of these data in this study, because there was no personal identifying information. Therefore, informed consent was also not required.

### Study population

**Figure 1:**
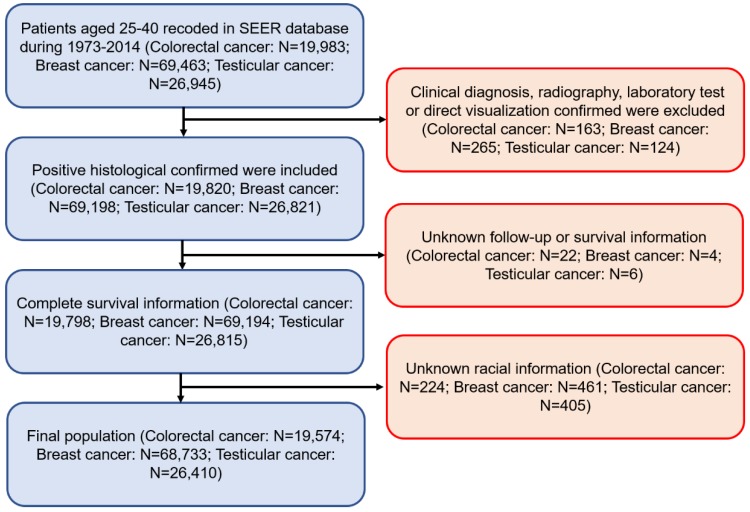
Flow chart of patient cohort selection.

Patients diagnosed with colorectal, breast and testicular cancer from 1973 to 2014 were included in this study (Figure 1). Primary cancer site and histology were coded according to criteria found in the third edition of the International Classification of Diseases for Oncology (ICD-O-3). All these participants classified as Whites, Blacks, and Others (American Indian/AK Native, Asian/Pacific Islander) and had a confirmed positive histology. The definition of a young patient with colorectal, breast and testicular cancer varies among previous studies. The upper age limit has ranged from 35 to 40 years 17, 18. Our study defined ≤40 years old as young-onset at preliminary diagnosis. Demographic and clinical information, such as gender, age, grade, cancer stage, distant metastasis, and therapies were extracted from the SEER database. Marital status was categorised as married, unmarried (divorced, separated, widowed, and single) or unknown. The primary outcomes were OS and cancer-specific survival (CSS) 19. OS was defined as the time from diagnosis to date of death from any cause. CSS was the interval between diagnosis and date of death from any of the listed cancers.

The exclusion criteria were as follows: (1) Patients diagnosed at autopsy or by death certificate, as well as patients with no histologically confirmed cancer. (2) Incomplete follow-up information or survival information. (3) Unknown racial information.

### Statistical analysis

The chi-square test was used for comparing tumour characteristics, treatment and mortality outcomes by race. Kaplan-Meier survival curves and the log-rank test were used to compare survival differences among races for the different tumours. In univariate and multivariate Cox proportional hazards models, survival was a function of sex, race, cancer stage at diagnosis, and treatments; hazard ratios (HRs) and 95% confidence intervals (CIs) were computed. A two-tailed P-value < 0.05 was considered significant. All statistical analyses were performed using IBM SPSS Statistics software (ver. 20.0; IBM Corp., Armonk, NY, USA), and figures were created using GraphPad Prism software (GraphPad Software, Inc., La Jolla, CA, USA).

## Results

### Patient characteristics

A total of 19,574 patients with colorectal cancer, 68,733 with breast cancer and 26,410 with testicular cancer were identified; the mean age of the groups was 34.4, 35.0 and 31.7 years, respectively. The baseline clinical characteristics of the patients are shown in Table 1. A higher proportion of Blacks presented with a distant stage at diagnosis compared to Whites and Others (colorectal cancer: 18.0%, 18.5% and 18.4%, respectively, P = 0.004; breast cancer: 3.5%, 6.3% and 4.0%, respectively, P < 0.001; testicular cancer: 6.9%, 10.8% and 8.6%, respectively, P < 0.001). In addition, Blacks with these tumours were less likely to be married (colorectal cancer: 59.0%, 37.8% and 60.6%, respectively, P < 0.001; breast cancer: 67.4%, 39.5% and 69.1%, respectively, P < 0.001; testicular cancer: 50.8%, 34.2% and 42.8%, respectively, P < 0.001). The proportion of Whites with breast cancer and testicular cancer who underwent surgery was significantly lower than in the Blacks and Others racial groups (colorectal cancer: 64.5%, 64.7% and 64.7%, respectively, P = 0.210; breast cancer: 60.6%, 66.0% and 69.3%, respectively, P < 0.001; testicular cancer: 65.7%, 72.0% and 69.1%, respectively, P < 0.001).

### Pattern of metastasis

Racial differences in the pattern of *de novo* distant metastasis likely contributed to the observed disparities among races in cancer survival. We explored the metastatic pattern of colorectal, breast and testicular cancers. Metastatic information was recorded in the SEER database after 2010, but only information about bone, brain, liver and lung were included at the time of diagnosis. In this cohort, 4,933 patients with colorectal cancer, 13,901 with breast cancer and 5,929 with testicular cancer were included to analyse the patterns of metastasis. We identified 1,346 (27.3%), 1,075 (7.7%) and 644 (10.9%) patients with colorectal, breast and testicular cancer, respectively, who had advanced stage disease at the time of diagnosis (Table 2). No significant difference in *de novo* metastasis was found between Blacks and Whites with colorectal cancer (P = 0.326). However, we observed significant racial differences in distant metastatic breast and testicular cancer: Blacks had higher rates of metastasis compared to the Whites and Others groups (breast cancer: 7.2%, 11.2%, and 6.4%, respectively, P < 0.001; testicular cancer: 10.7%, 16.7%, and 10.0%, respectively, P = 0.036).

The pattern of *de novo* metastasis within the primary sites is presented in Table 2. Among patients with colorectal cancer, the most common metastatic site was the liver (16.8%, 18.8% and 14.8% for Whites, Blacks and Others, respectively, P = 0.281). Among patients with breast cancer, the most common metastatic site was the bone (4.5%, 6.1% and 3.7% for Whites, Blacks and Others, respectively, P < 0.001). The lung was the most common metastatic site for patients with testicular cancer (6.7%, 10.6% and 6.1% for Whites, Blacks and Others, respectively, P = 0.237).

### Survival analysis

**Figure 2:**
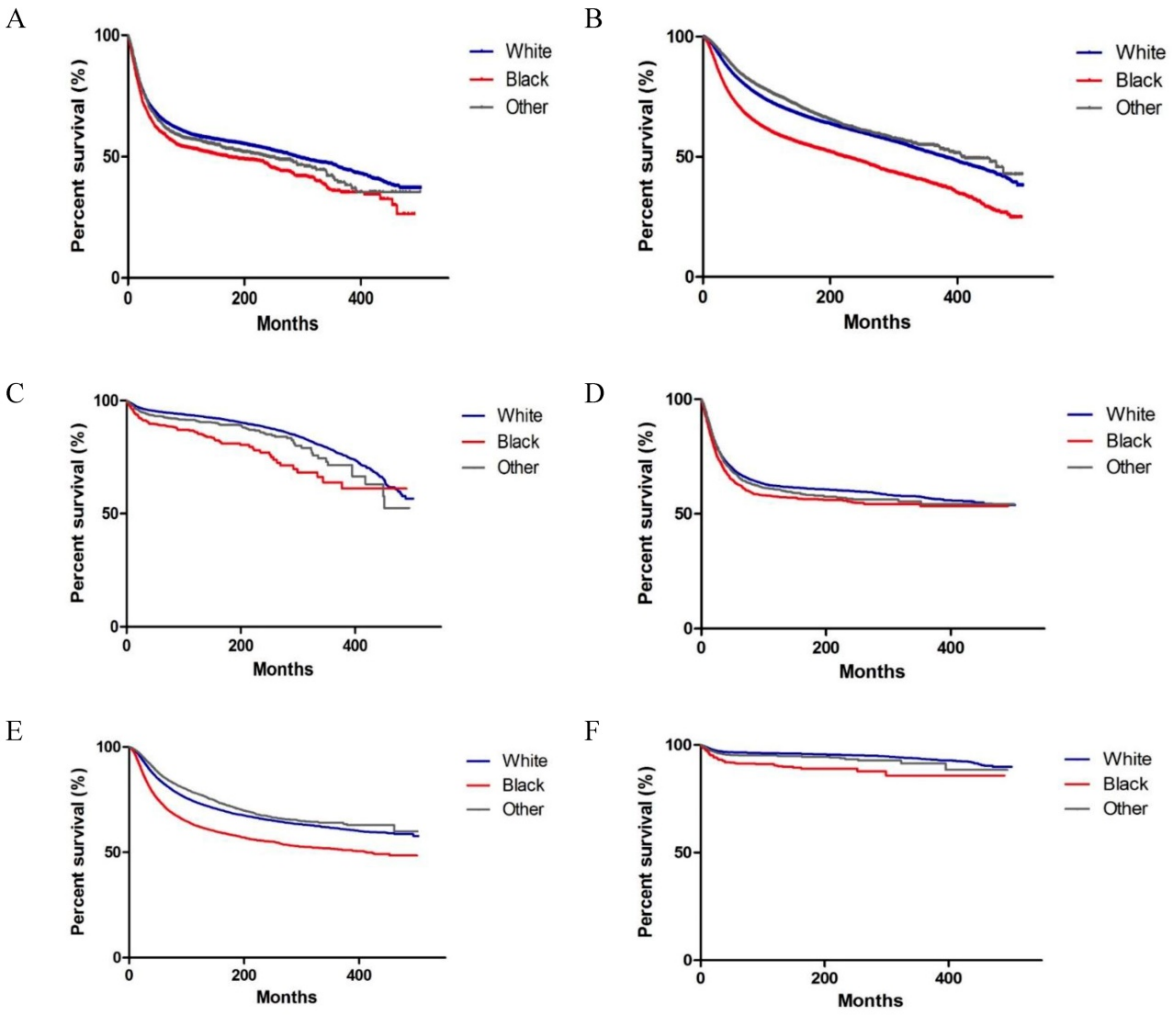
Kaplan-Meier survival curves of survival according to race (Whites, Blacks, and Others) in patients with colorectal, breast and testicular cancer. (A-C) Overall survival of colorectal, breast and testicular cancer. (D-F) Cancer-specific survival of colorectal, breast, and testicular cancer.

As shown in Figure 2, significant differences were observed in OS and CSS among Whites, Blacks and Others with colorectal, breast and testicular cancer (both P < 0.001 on log-rank test). We performed a multivariate analysis of the impact of race on mortality, while adjusting for prognostic factors influencing survival (Tables 3 and 4). We observed that Blacks had the highest overall mortality rate (colorectal cancer, HR, 1.277, 95% CI: 1.198, 1.361, P < 0.001; breast cancer, HR, 1.471, 95% CI: 1.420, 1.525, P < 0.001; testicular cancer, HR, 1.887, 95% CI: 1.562, 2.281, P < 0.001) (Table 3). Similar results were found when CSS was analysed (Table 4). In addition to race, other variables, such as gender (for colorectal cancer), year, marital status, grade (for colorectal cancer and breast cancer), stage, surgery, chemotherapy, and radiation (for colorectal cancer) were identified as prognostic factors.

### Relationship between race and survival in patients with colorectal, breast and testicular cancer by marital status, surgery and disease stage

According to the results from multivariate Cox proportional hazards models, marital status, surgery and disease stage affect the prognosis of these cancers. We further stratified the analysis of overall mortality by marital status, surgery and disease stage (Table 5). The influence of race on OS did not differ by marital status, surgery and disease stage. For colorectal cancer, Blacks, particularly those who were unmarried were more likely to have inferior OS (HR, 1.318, 95% CI: 1.211, 1.435, P < 0.001). The HR of unmarried Blacks was 1.465 for breast cancer (HR, 1.465, 95% CI: 1.394, 1.541, P < 0.001). Unmarried Blacks had the highest HR for testicular cancer (HR, 1.944, 95% CI: 1.544, 2.447, P < 0.001). Among patients who accepted cancer-directed surgery, Blacks had a higher HR than Whites and Others (colorectal cancer, HR, 1.372, 95% CI: 1.253, 1.503, P < 0.001; breast cancer; HR, 1.488, 95% CI: 1.410, 1.571, P < 0.001; testicular cancer, HR, 1.924, 95% CI: 1.426, 2.597, P < 0.001). The effect of race on mortality was weak among those who did not accept surgery for colorectal cancer (Blacks, HR, 1.027, 95% CI: 0.866, 1.219, P = 0.758; Others, HR, 0.919, 95% CI: 0.760, 1.112, P = 0.386) and testicular cancer (Blacks, HR, 1.039, 95% CI: 0.538, 2.007, P = 0.909; Others, HR, 0.772, 95% CI: 0.388, 1.533, P = 0.459). In subgroup analysis of colorectal and breast cancer, Blacks had a higher risk of mortality than Whites at every disease stage, with greatest disparities noted among individuals with localized stage.

## Discussion

Previously studies demonstrated that Blacks had significantly higher risk of mortality compared with Whites regardless of primary cancer type 16, 20. Our study confirmed that Blacks appear to have worse survival than Whites for primary young-onset colorectal, breast and testicular cancer (colorectal cancer, HR, 1.277, 95% CI: 1.198, 1.361, P < 0.001; breast cancer, HR, 1.471, 95% CI: 1.420, 1.525, P < 0.001; testicular cancer, HR, 1.887, 95% CI: 1.562, 2.281, P < 0.001). We observed racial differences in survival at all disease stages in patients with colorectal cancer or breast cancer, with the greatest disparities noted among individuals with localized disease stage at the time of diagnosis. *De novo* metastasis was most prevalent among patients with primary colorectal cancer (27.3%), followed by testicular cancer (10.9%) and breast cancer (7.7%). And the most common site of these cancer types were liver, lung and bone respectively, which was consistent with other studies 16. We observed significant differences in distant metastases of breast and testicular cancer among races. Blacks had a higher rate of metastasis at the time of diagnosis compared to Whites and Others (breast cancer: 11.2%, 7.2%, and 6.4%, respectively, P < 0.001; testicular cancer: 16.7%, 10.7%, and 10.0%, respectively, P = 0.036). However, no significant difference in *de novo* metastasis was found between Blacks and Whites for colorectal cancer (P = 0.326). In the current study, there were several notable and interesting findings. First, the HR of mortality was highest among unmarried Blacks with colorectal, breast and testicular cancers (colorectal cancer: HR, 1.318, 95% CI: 1.211, 1.435, P < 0.001; breast cancer: HR, 1.465, 95% CI: 1.394, 1.541, P < 0.001; testicular cancer: HR, 1.944, 95% CI: 1.544, 2.447, P < 0.001). It is reasonable to assume that married patients have greater financial resources, due to higher income and better employment, as well as health insurance, which ultimately influences the likelihood of early diagnosis and timely medical care. Second, our results differ from previous studies in that the frequency of surgery was significantly lower among Whites with breast cancer and testicular cancer versus Blacks and Others (colorectal cancer: 64.5%, 64.7% and 64.7%, respectively, P = 0.210; breast cancer: 60.6%, 66.0% and 69.3%, respectively, P < 0.001; testicular cancer: 65.7%, 72.0% and 69.1%, respectively, P < 0.001), partly because young White patients with breast or testicular cancer have higher rates of refusing a mammectomy or testectomy. Third, an influence of race on OS was notable among patients who accepted cancer-directed surgery. The effect of race on mortality was weak among those who did not accept surgery for colorectal cancer and testicular cancer. These novel findings raised a number of questions about the possible impact that treatment factors and patient factors have with regard to differences in survival by race. Previous studies have demonstrated that upstream factors, such as access to healthcare, routine screening, racial bias and socioeconomic status, contribute to the late-stage diagnosis and higher mortality experienced by Black patients with cancer 21, 22. Our study supports the conclusions of previous studies about racial differences in stage at diagnosis, which may be partially due to racial differences in access to routine healthcare and routine cancer screening, and by extension early detection of cancer. Access to primary care is lower among Blacks than Whites. Even among adults with access to primary care, care for Black patients is largely provided by a subset of US healthcare providers who are less well-trained 23, 24. Although it is widely accepted that differences in quality of care contribute to racial disparities in survival outcomes, some findings have suggested that equal treatments may not necessarily result in equal outcomes 20. As shown in the multivariate analysis, Black patients had worse OS and CSS than the other racial groups, even after adjusting for sex, marital status, age, grade, and therapies. We hypothesised that many biological factors lead to racial differences in the health of general populations, including molecular, biochemical, cellular, and/or genetic factors that might predispose Blacks to faster growing, metastasising tumours 19, 25.

Several potential limitations to our study should be discussed. First, the racial and geographic distributions of patients in the SEER 18 registries differed from that of the entire US population. Blacks represent 10.9% of SEER patients compared to 12.6% of the US population, and 21.6% of SEER patients reside in the South compared to 37.1% of the US population 8, 23. Thus, sampling bias may have affected the results. In addition, the socioeconomic status of the patients influences the cancer prognosis, but a lack of data inhibited our ability to examine its effect on the association of race with survival outcomes. Furthermore, treatment modalities were largely limited to surgery, chemotherapy, and radiation, although treatment modality is known to be an important factor associated with racial disparities in cancer survival rates.

## Conclusion

Our study demonstrated that Black patients with colorectal, breast and testicular cancer, particularly those unmarried at the time of diagnosis, had worse OS compared to other races. Especially, Black patients with colorectal and breast cancer had a poorer prognosis at all disease stage compared with White counterparts. The risk of mortality remained to be higher among Black patients who had accepted cancer-directed surgery. However, the effect of race was attenuated among patients who did not undergo surgery for colorectal and testicular cancer. Addressing racial disparities in survival requires more than just equalising access to surgical resection; equal access to high-quality medical care, greater social support and more comprehensive interventions are required. Further studies of the clinical and molecular characteristics of young-onset colorectal, breast and testicular cancer are needed to explore the racial differences in survival and to refine clinical algorithms for treatment and early detection.

## Figures and Tables

**Table 1:** Patient sociodemographic and clinical characteristics by race (SEER 1973-2014). Data are percent.

Variables	Colorectal cancer n = 19,574			Breast cancer n = 68,733			Testicular cancer n = 26,410		
	White 75.1	Black 13.7	Other 11.2	White 74.7	Black 15.0	Other 10.3	White 92.8	Black 2.7	Other 4.6
Sex									
Male	**53.0**	**45.5**	**52.4**	-	-	-	-	-	-
Female	**47.0**	**54.5**	**47.6**	-	-	-	-	-	-
Year									
1973-1993	**20.9**	**21.4**	**16.8**	**28.3**	**23.3**	**17.6**	**24.2**	**16.1**	**19.3**
1994-2014	**79.1**	**78.6**	**83.2**	**71.7**	**76.7**	**82.4**	**75.8**	**83.9**	**80.7**
Married status									
Married	**59.0**	**37.8**	**60.6**	**67.4**	**39.5**	**69.1**	**50.8**	**34.2**	**42.8**
Unmarried	**36.7**	**56.4**	**34.8**	**29.3**	**56.3**	**27.4**	**45.2**	**59.8**	**52.2**
Unknown	**4.4**	**5.8**	**4.6**	**3.3**	**4.2**	**3.4**	**4.0**	**6.0**	**5.0**
Grade									
Well	**9.7**	**10.2**	**8.1**	**5.6**	**3.7**	**7.5**	0.7	1.1	0.6
Moderately	**48.6**	**49.4**	**48.5**	**24.9**	**20.5**	**29.1**	0.4	0.1	0.8
Poor	**19.1**	**15.9**	**23.9**	**43.9**	**51.6**	**44.5**	1.2	1.8	1.1
Undifferentiated	**2.3**	**1.3**	**1.3**	**2.8**	**2.3**	**2.3**	2.6	2.6	2.5
unknown	**20.4**	**23.2**	**18.2**	**22.8**	**21.9**	**16.6**	95.2	94.3	95.0
Stage									
Localised	**23.1**	**23.1**	**21.8**	**30.3**	**28.4**	**37.9**	**48.0**	**46.4**	**51.1**
Regional	**29.0**	**26.2**	**31.3**	**28.7**	**32.1**	**30.8**	**11.8**	**17.3**	**11.4**
Distant	**18.0**	**18.5**	**18.4**	**3.5**	**6.3**	**4.0**	**6.9**	**10.8**	**8.6**
Unknown	**29.9**	**32.2**	**28.5**	**37.5**	**33.2**	**27.3**	**33.4**	**25.4**	**28.9**
Surgery									
Yes	64.5	64.7	64.7	**60.6**	**66.0**	**69.3**	**65.7**	**72.0**	**69.1**
No	8.6	9.6	9.5	**3.9**	**5.9**	**4.8**	**1.5**	**3.7**	**2.7**
Unknown	26.9	25.6	25.8	**35.5**	**28.1**	**25.9**	**32.7**	**24.3**	**28.3**
Radiation									
Yes	**20.3**	**16.7**	**22.7**	**42.5**	**42.6**	**45.3**	32.7	28.6	32.2
No/unknown	**79.7**	**83.3**	**77.3**	**57.5**	**57.4**	**54.7**	67.3	71.4	67.8
Chemotherapy									
Yes	**53.0**	**47.0**	**57.6**	**64.7**	**66.0**	**66.5**	28.6	30.5	31.1
No/unknown	**47.0**	**53.0**	**42.4**	**35.3**	**34.0**	**33.5**	71.4	69.5	68.9

Bold indicates level of statistical significance achieved at α =0.05. Other: American Indian/AK Native, Asian/Pacific Islander.

**Table 2:** Pattern of *de novo* metastasis by race and primary site (SEER 2010-2014).

Any metastasis	Race		
	White	Black	Other
Colorectal cancer (n = 4,933)			
No metastasis	2719(72.8%)	465(70.7%)	405(74.6%)
Any metastasis	1014(27.2%)	193(29.3%)	139(25.6%)
To liver	626(16.8%)	124(18.8%)	80(14.8%)
To bone	46(1.2%)	12(1.8%)	11(2.0%)
To brain	9 (0.2%)	2 (0.3%)	1 (0.2%)
To lung	166 (4.4%)	40 (6.1%)	30 (5.5%)
Breast cancer (n = 13,901)			
No metastasis	**9,147 (92.8%)**	**1,950 (88.8%)**	**1,729 (93.6%)**
Any metastasis	**709 (7.2%)**	**247 (11.2%)**	**119 (6.4%)**
To liver	**226 (2.3%)**	**89 (4.1%)**	**36 (1.9%)**
To bone	**448 (4.5%)**	**134 (6.1%)**	**69 (3.7%)**
To brain	47 (0.5%)	16 (0.7%)	8 (0.4%)
To lung	**147 (1.5%)**	**77 (3.5%)**	**21 (1.1%)**
Testicular cancer (n = 5,929)			
No metastasis	**4,811 (89.3%)**	**150 (83.3%)**	**324 (90.0%)**
Any metastasis	**578 (10.7%)**	**30 (16.7%)**	**36 (10.0%)**
To liver	90 (1.7%)	7 (3.9%)	8 (2.2%)
To bone	41 (0.8%)	2 (1.1%)	0 (0.0%)
To brain	43 (0.8%)	0 (0.0%)	2 (0.6%)
To lung	361 (6.7%)	19 (10.6%)	22 (6.1%)

Bold indicates level of statistical significance achieved at α = 0.05. Other: American Indian/AK Native, Asian/Pacific Islander.

**Table 3:** Multivariate analyses of overall survival in patients with colorectal, breast and testicular cancer in the SEER database.

Variables	Primary sites					
	Colorectal cancer OS HR (95% CI)	P-value	Breast cancer OS HR (95% CI)	P-value	Testicular cancer OS HR (95% CI)	P-value
Sex						
Male	Reference					
Female	0.794 (0.759, 0.831)	<0.001	-		-	
Year						
1973-1993	Reference		Reference		Reference	
1994-2014	0.739 (0.678, 0.805)	<0.001	0.799 (0.762, 0.839)	<0.001	0.598 (0.515,0.696)	<0.001
Married status						
Married	Reference		Reference		Reference	
Unmarried	1.238 (1.181, 1.297)	<0.001	1.162 (1.128, 1,197)	<0.001	1.665 (1.538, 1.803)	<0.001
Unknown	1.055 (0.932, 1.193)	0.397	1,072 (0.990, 1.161)	0.086	1.536 (1.233, 1.912)	<0.001
Grade						
Well	Reference		Reference		Reference	
Moderately	1.299 (1.181,1.429)	<0.001	1.707 (1.538, 1.895)	<0.001	1.449 (0.731, 2.872)	0.288
Poor	2.096 (1.897,2.316)	<0.001	2.362 (2.135, 2.615)	<0.001	1.697 (0.982, 2.933)	0.058
Undifferentiated	2.914 (2.484,3.418)	<0.001	2.536 (2.244, 2.866)	<0.001	1.465 (0.874, 2.457)	0.147
unknown	1.324 (1.195,1.467)	<0.001	2.206 (1.989, 2.446)	<0.001	1.188 (0.726, 1.944)	0.494
Stage						
Localised	Reference		Reference		Reference	
Regional	2.443 (2.179, 2.740)	<0.001	2.498 (2.372, 2.630)	<0.001	1.688 (1.373, 2.026)	<0.001
Distant	10.358 (9.249, 11.600)	<0.001	9.326 (8.674, 10.027)	<0.001	6.374 (5.392, 7.535)	<0.001
Unknown	3.572 (3.062, 4.167)	<0.001	2.652 (2.430, 2.895)	<0.001	2.719 (1.655, 4.467)	<0.001
Surgery						
Yes	Reference		Reference		Reference	
No	2.219 (2.059, 2.392)	<0.001	2.166 (2.019, 2.322)	<0.001	2.228 (1.830, 2.741)	<0.001
Unknown	1.258 (1.101, 1.438)	0.001	1.055 (0.974, 1.142)	0.187	0.776 (0.469, 1.284)	0.324
Radiation						
Yes	Reference		Reference		Reference	
No/unknown	0.923 (0.873, 0.976)	0.005	1.017 (0.988, 1.047)	0.253	0.960 (0.873, 1.056)	0.405
Chemotherapy						
Yes	Reference		Reference		Reference	
No/unknown	0.696 (0.656, 0.737)	<0.001	0.717 (0.694, 0.741)	<0.001	0.512 (0.464, 0.565)	<0.001
Race						
White	Reference		Reference		Reference	
Black	1.277 (1.198, 1.361)	<0.001	1.471 (1.420, 1.525)	<0.001	1.887 (1.562,2.281)	<0.001
Other	1.004 (0.934, 1.079)	0.911	0.934 (0.887, 0.983)	0.008	1.218 (1.021, 1.452)	0.028

Abbreviations: OS, overall survival; HR, hazard ratio; CI, confidence interval. Other: American Indian/AK Native, Asian/Pacific Islander. Adjusted for gender (only for colorectal cancer), year, grade, stage, therapy, marital status and race.

**Table 4:** Multivariate analyses of cancer-specific survival in patients with colorectal, breast and testicular cancer in the SEER database (1973-2014).

Variables	Primary sites					
	Colorectal cancer CSS HR (95% CI)	P-value	Breast cancer CSS HR (95% CI)	P-value	Testicular cancer CSS HR (95% CI)	P-value
Sex						
Male	Reference					
Female	1.229 (1.170, 1.291)	<0.001	-		-	
Year						
1973-1993	Reference		Reference		Reference	
1994-2014	0.696 (0.634, 0.764)	<0.001	0.786 (0.746, 0.827)	<0.001	0.532 (0.423, 0.669)	<0.001
Married status						
Married	Reference		Reference		Reference	
Unmarried	1.187 (1.128, 1.249)	<0.001	1.128 (1.093, 1.165)	<0.001	1.518 (1.346, 1.713)	<0.001
Unknown	0.954 (0.830, 1.096)	0.506	1,018 (0.932, 1.111)	0.697	1.582 (1.155, 2.168)	0.004
Grade						
Well	Reference		Reference		Reference	
Moderately	1.353 (1.216, 1.506)	<0.001	1.874 (1.665, 2.110)	<0.001	1.426 (0.506, 4.017)	0.501
Poor	2.215 (1.983, 2.474)	<0.001	2.628 (2.341, 2.949)	<0.001	1.365 (0.579, 3.218)	0.477
Undifferentiated	2.977 (2.505, 3.538)	<0.001	2.815 (2.453, 3.230)	<0.001	1.059 (0.455, 2.466)	0.894
unknown	1.341(1.195, 1.504)	<0.001	2.444 (2.173, 2.750)	<0.001	0.935 (0.418, 2.089)	0.869
Stage						
Localised	Reference		Reference		Reference	
Regional	3.096 (2.698, 3.553)	<0.001	2.719 (2.570, 2.876)	<0.001	1.759 (1.294, 2.390)	<0.001
Distant	13.969 (12.197, 15.999)	<0.001	10.432 (9.660, 11.265)	<0.001	10.722 (8.388, 13.707)	<0.001
Unknown	4.629 (3.872, 5.533)	<0.001	2.976 (2.710, 3.269)	<0.001	5.027 (2.736, 9.236)	<0.001
Surgery						
Yes	Reference		Reference		Reference	
No	2.226 (2.058, 2.408)	<0.001	2.155 (2.002, 2.318)	<0.001	2.267 (1.818, 2.827)	<0.001
Unknown	1.276 (1.103, 1.476)	0.001	1.039 (0.955, 1.130)	0.379	0.709 (0.384, 1.307)	0.271
Radiation						
Yes	Reference		Reference		Reference	
No/unknown	0.923 (0.870, 0.979)	0.008	1.028 (0.997, 1.061)	0.079	1.064 (0.902, 1.255)	0.461
Chemotherapy						
Yes	Reference		Reference		Reference	
No/unknown	0.632 (0.593, 0.674)	<0.001	0.670 (0.647, 0.695)	<0.001	0.299 (0.256, 0.349)	<0.001
Race						
White	Reference		Reference		Reference	
Black	1.251 (1.167, 1.341)	<0.001	1.459 (1.404, 1.517)	<0.001	2.172 (1.692, 2.788)	<0.001
Other	0.985 (0.912, 1.065)	0.710	0.919 (0.870, 0.971)	0.003	1.237 (0.963, 1.589)	0.096

Abbreviations: CSS, cancer-specific survival; HR, hazard ratio; CI, confidence interval. Other: American Indian/AK Native, Asian/Pacific Islander. Adjusted for gender (only for colorectal cancer), year, grade, stage, therapy, marital status and race.

**Table 5:** Adjusted HRs for OS by race (Whites as reference, SEER 1973-2014).

Variables	Colorectal cancer OS HR (95% CI)		Breast cancer OS HR (95% CI)		Testicular cancer OS HR (95%CI)	
	Black	Other	Black	Other	Black	Other
Marital status^#^						
Married	**1.176 (1.059,1.305)**	1.021 (0.930,1.122)	**1.453 (1.377,1.533)**	0.963 (0.905,1.024)	**1.775 (1.239,2.543)**	1.084 (0.795,1.478)
Unmarried	**1.318 (1.211,1.435)**	0.957 (0.850,1.078)	**1.465 (1.394,1.541)**	**0.887 (0.807,0.975)**	**1.944 (1.544,2.447)**	**1.271 (1.017,1.588)**
Unknown	**1.551 (1.140,2.110)**	1.252 (0.865,1.811)	**1.535 (1.280,1.842)**	0.799 (0.579,1.103)	1.454 (0.567,3.729)	1.286 (0.581,2.847)
Surgery*						
Yes	**1.372 (1.253,1.503)**	1.082 (0.976,1.200)	**1.488 (1.410,1.571)**	0.942 (0.872,1.019)	**1.924 (1.426,2.597)**	1.194 (0.877,1.625)
No	1.027 (0.866,1.219)	0.919 (0.760,1.112)	**1.471 (1.292,1.675)**	0.963 (0.795,1.166)	1.039 (0.538,2.007)	0.772 (0.388,1.533)
Unknown	**1.233 (1.110,1.370)**	0.943 (0.837,1.062)	**1.457 (1.384,1.534)**	0.931 (0.867,1.001)	**2.044 (1.572,2.658)**	**1.349 (1.077,1.690)**
Stage^&^						
Localized	**1.372(1.184,1.590)**	**1.210(1.040,1.408)**	**1.524(1.421,1.634)**	0.957(0.867,1.057)	1.697(0.913,3.135)	**2.062(1.140,3.727)**
Regional	**1.354(1.046,1.753)**	0.841(0.589,1.201)	**1.514(1.356, 1.690)**	0.891(0.768,1.034)	**2.149(1.312,3.520)**	1.301(0.796,2.216)
Distant	**1.148(1.025,1.285)**	0.974(0.860,1.103)	**1.402(1.252,1.570)**	0.937(0.792,1.108)	1.455(0.985,2.148)	0.826(0.542,1.259)
Unknown	**1.264(1.148,1.393)**	0.935 (0.833,1.049)	**1.452(1.382,1.525)**	0.938(0.874,1.006)	**1.985(1.526,2.581)**	**1.332(1.063,1.668)**

#Adjusted for gender (only for colorectal cancer), year, grade, stage, therapy (surgery, radiation and chemotherapy), and race. *Adjusted for gender (only for colorectal cancer), year, grade, stage, therapy (radiation and chemotherapy), race, and marital status. & Adjusted for gender (only for colorectal cancer), year, grade, therapy (surgery, radiation and chemotherapy), race, and marital status. Bold indicates significance at α = 0.05. Abbreviation: OS, overall survival; HR, hazard ratio; CI, confidence interval.
